# The genome sequence of the hawthorn leaf beetle, *Lochmaea crataegi *(Forster, 1771)

**DOI:** 10.12688/wellcomeopenres.20911.1

**Published:** 2024-02-19

**Authors:** Liam M. Crowley, Mark G. Telfer, Hermes E. Escalona, University of Oxford and Wytham Woods Genome Acquisition Lab, Darwin Tree of Life Barcoding collective, Wellcome Sanger Institute Tree of Life Management, Samples and Laboratory team, Wellcome Sanger Institute Scientific Operations: Sequencing Operations, Wellcome Sanger Institute Tree of Life Core Informatics team, Tree of Life Core Informatics collective, Darwin Tree of Life Consortium

**Affiliations:** 1 University of Oxford, Oxford, England, UK; 2 Independent researcher, Entomological Consultant, Ventnor, Isle of Wight, England, UK; 3 Australian National Insect Collection, CSIRO Consortium, Canberra, Australia

**Keywords:** Lochmaea crataegi, hawthorn leaf beetle, genome sequence, chromosomal, Coleoptera

## Abstract

We present a genome assembly from an individual male *Lochmaea crataegi* (the hawthorn leaf beetle; Arthropoda; Insecta; Coleoptera; Chrysomelidae). The genome sequence is 891.3 megabases in span. Most of the assembly is scaffolded into 16 chromosomal pseudomolecules, including the X and Y sex chromosomes. The mitochondrial genome has also been assembled and is 18.32 kilobases in length.

## Species taxonomy

Eukaryota; Opisthokonta; Metazoa; Eumetazoa; Bilateria; Protostomia; Ecdysozoa; Panarthropoda; Arthropoda; Mandibulata; Pancrustacea; Hexapoda; Insecta; Dicondylia; Pterygota; Neoptera; Endopterygota; Coleoptera; Polyphaga; Cucujiformia; Chrysomeloidea; Chrysomelidae; Galerucinae; Galerucini; *Lochmaea*; *Lochmaea crataegi* ( Forster, 1771) (NCBI:txid1143063).

## Background

The Hawthorn Leaf Beetle, *Lochmaea crataegi,* is a distinctive leaf beetle about 4–5 mm long. It can be recognised by the glabrous dorsal surface, reddish colour, four elongate black marks on the elytra and pair of black spots on pronotum. The taxonomic history of the species is complex, with at least six junior synonyms with type localities in Central Europe ( Bezděk, 2004).

*Lochmaea* Weise, 1883 is a genus of leaf beetle widely distributed in Europe and Asia with about a dozen species divided in groups or subgenera. They are phytophagous beetles that use several plant families as host, including Betulaceae, Salicaceae, Rosaceae, Fagaceae, Ericaceae, and Cucurbitaceae ( Jolivet & Hawkeswood, 1995). The species *Lochmaea crataegi* ( Forster, 1771) is broadly distributed in Europe, Siberia and east Asia ( Bezděk, 2004; Warchałowski, 2003) with recent records for Taiwan ( Lee, 2019). The species was originally described from England by the pastor and naturalist Johann Reinhold Forster (1729–1798).

As the common name suggests, it is associated with common hawthorn, *Crataegus monogyna* Jacq. (Rosaceae), with both adults and larvae feeding on it and in some cases affecting the plant’s viability ( Bezděk, 2004; Warchałowski, 2003). Adults appear shortly before the flowers open, upon which time they begin feeding on the pollen, occasionally feeding on pollen of other species. The larvae feed exclusively on the developing fruits, developing throughout the summer before dropping to the soil to pupate. Adults eclose during the late summer to autumn and overwinter in this stage. It occurs across a wide range of habitats, anywhere where the host may be found.

The genomic information on *L. crataegi* will allow to disentangle the taxonomy and systematics of the species as well as the genus and promote insect-host adaptation studies, being the insect and the host both common and widely distributed in Europe.

## Genome sequence report

**Figure 1.**
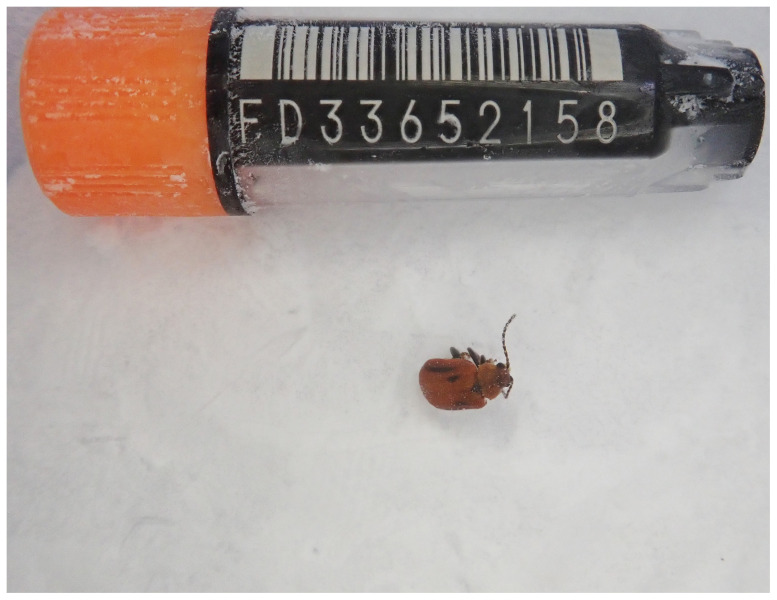
Photograph of the *Lochmaea crataegi* (icLocCrat2) specimen used for genome sequencing.

The genome was sequenced from one male *Lochmaea crataegi* ( Figure 1) collected from | Wytham Woods, Oxfordshire, UK (51.77, –1.34). A total of 35-fold coverage in Pacific Biosciences single-molecule HiFi long reads was generated. Primary assembly contigs were scaffolded with chromosome conformation Hi-C data. Manual assembly curation corrected 66 missing joins or mis-joins and removed 8 haplotypic duplications, reducing the assembly length by 0.45% and the scaffold number by 29.38%.

**Figure 2.**
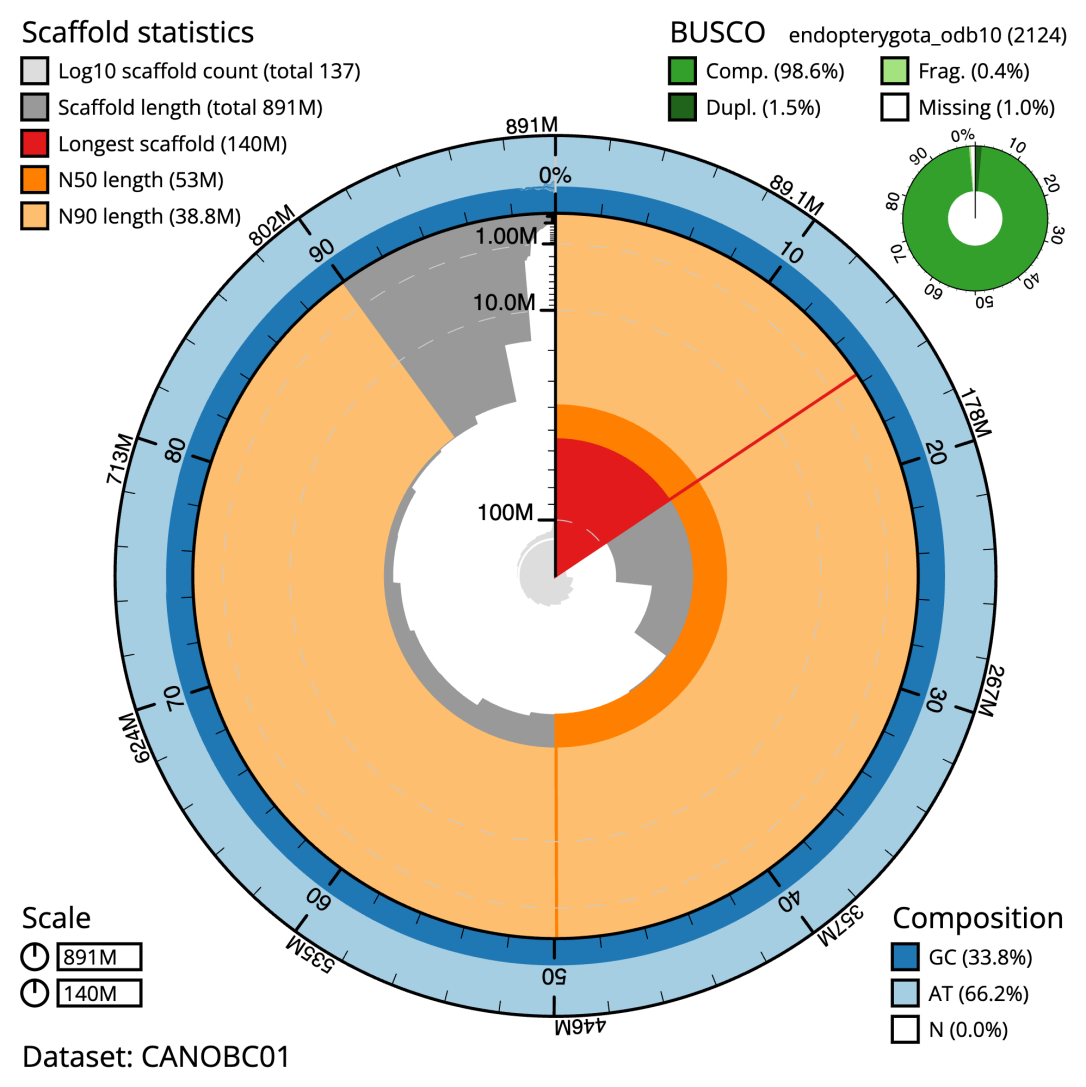
Genome assembly of *Lochmaea crataegi*, icLocCrat2.1: metrics. The BlobToolKit Snailplot shows N50 metrics and BUSCO gene completeness. The main plot is divided into 1,000 size-ordered bins around the circumference with each bin representing 0.1% of the 891,301,393 bp assembly. The distribution of scaffold lengths is shown in dark grey with the plot radius scaled to the longest scaffold present in the assembly (139,762,297 bp, shown in red). Orange and pale-orange arcs show the N50 and N90 scaffold lengths (53,024,378 and 38,778,232 bp), respectively. The pale grey spiral shows the cumulative scaffold count on a log scale with white scale lines showing successive orders of magnitude. The blue and pale-blue area around the outside of the plot shows the distribution of GC, AT and N percentages in the same bins as the inner plot. A summary of complete, fragmented, duplicated and missing BUSCO genes in the endopterygota_odb10 set is shown in the top right. An interactive version of this figure is available at https://blobtoolkit.genomehubs.org/view/CANOBC01/dataset/CANOBC01/snail.

**Figure 3.**
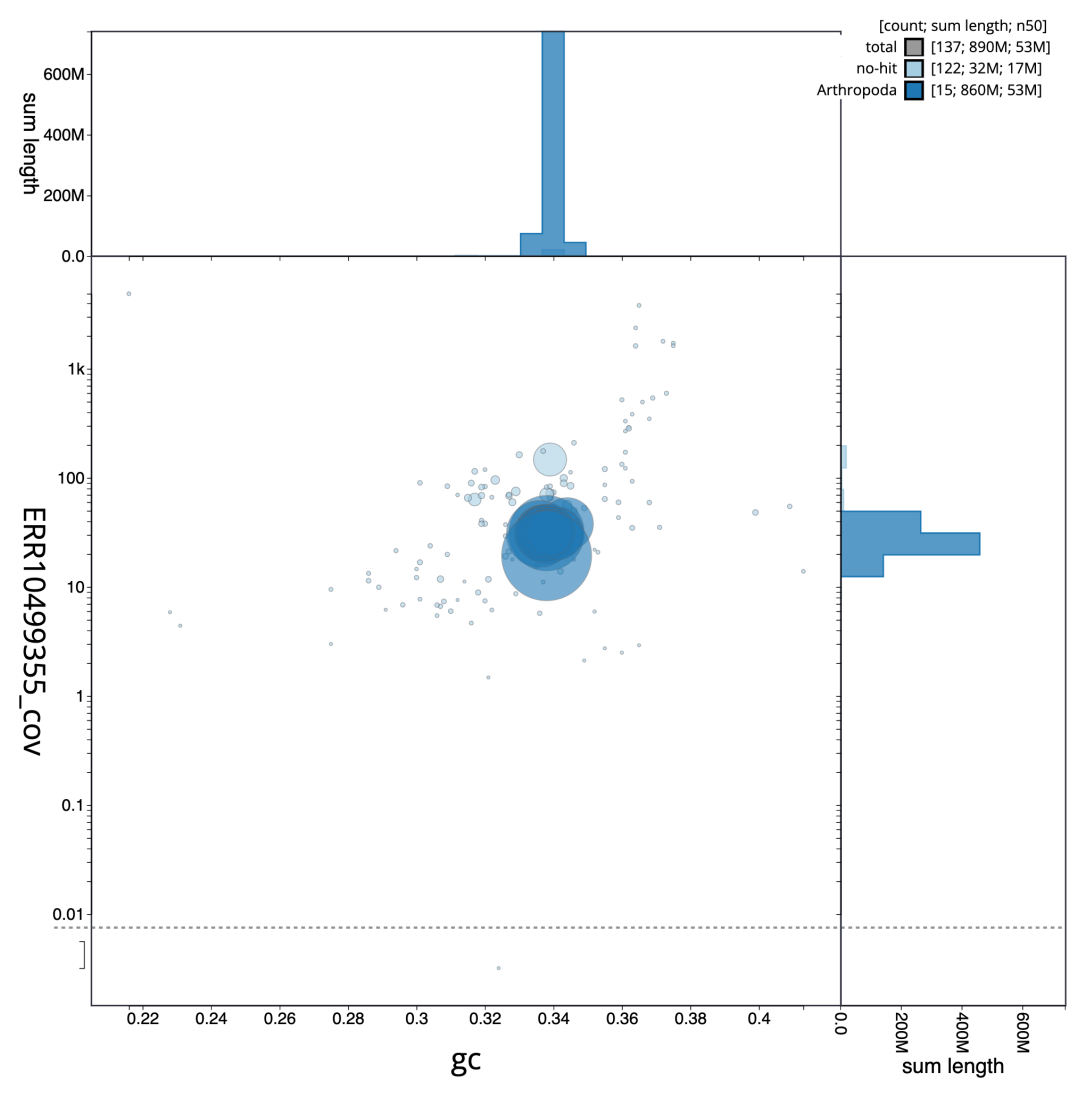
Genome assembly of *Lochmaea crataegi*, icLocCrat2.1: BlobToolKit GC-coverage plot. Sequences are coloured by phylum. Circles are sized in proportion to sequence length. Histograms show the distribution of sequence length sum along each axis. An interactive version of this figure is available at https://blobtoolkit.genomehubs.org/view/CANOBC01/dataset/CANOBC01/blob.

**Figure 4.**
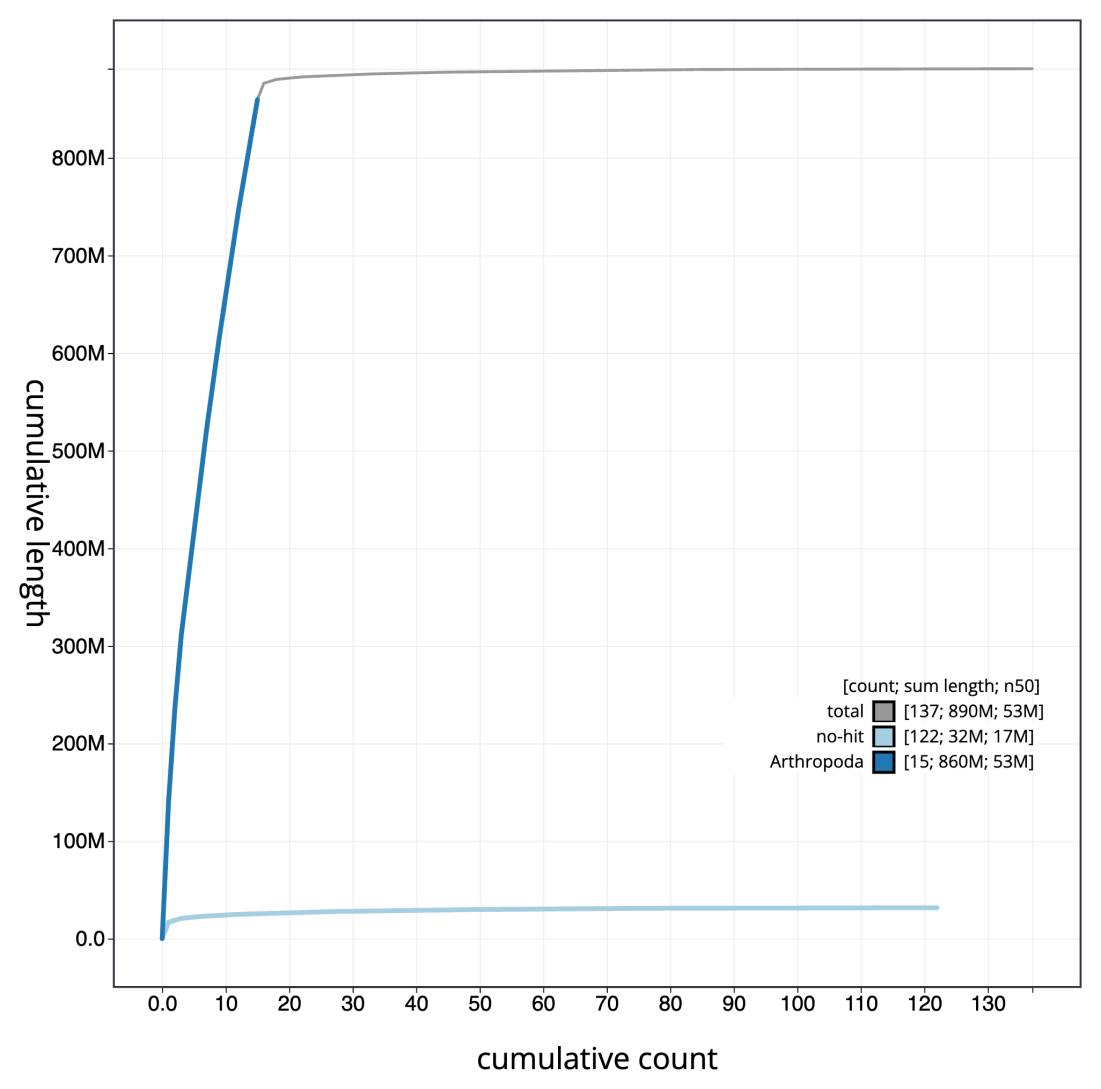
Genome assembly of *Lochmaea crataegi*, icLocCrat2.1: BlobToolKit cumulative sequence plot. The grey line shows cumulative length for all sequences. Coloured lines show cumulative lengths of sequences assigned to each phylum using the buscogenes taxrule. An interactive version of this figure is available at https://blobtoolkit.genomehubs.org/view/CANOBC01/dataset/CANOBC01/cumulative.

**Figure 5.**
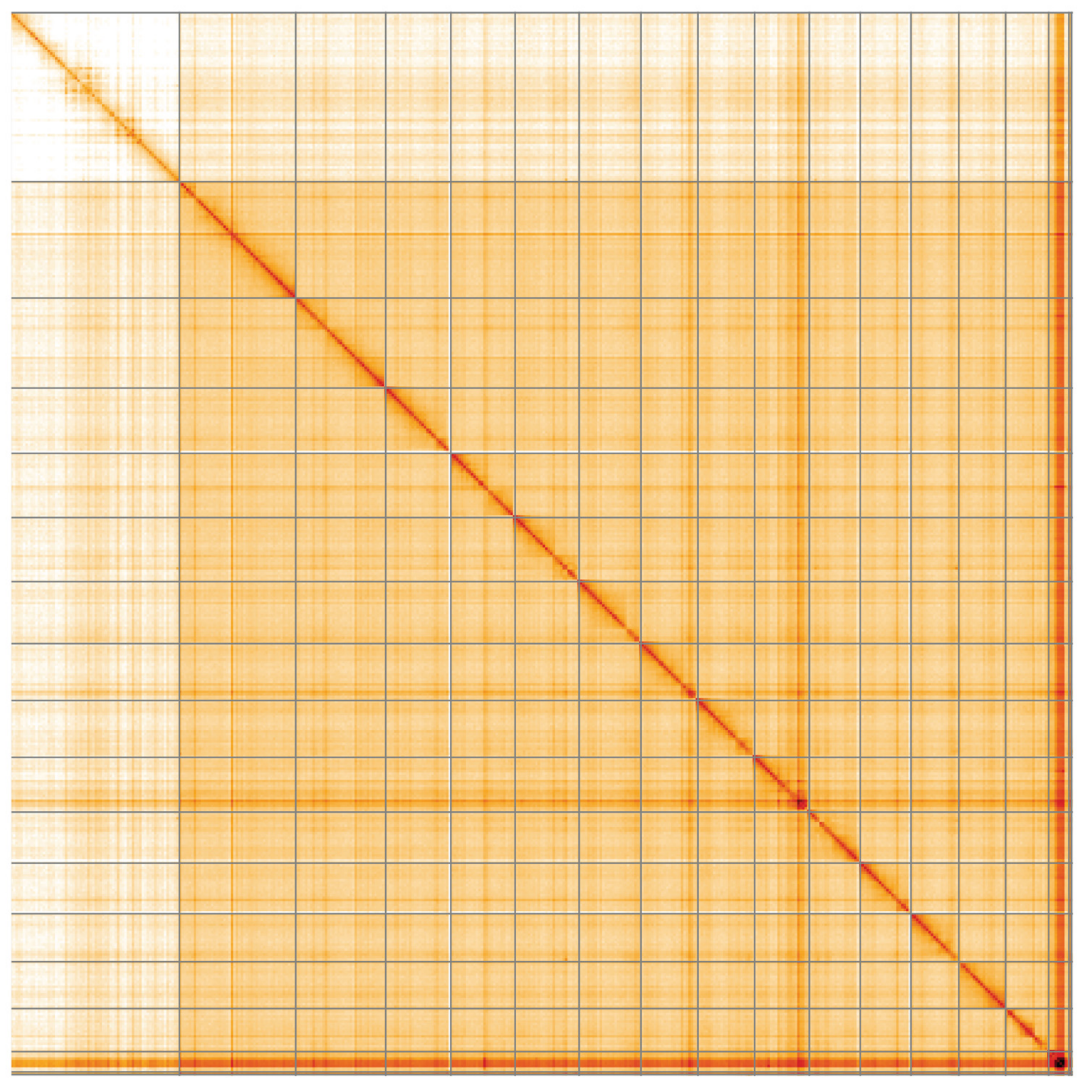
Genome assembly of *Lochmaea crataegi*, icLocCrat2.1: Hi-C contact map of the icLocCrat2.1 assembly, visualised using HiGlass. Chromosomes are shown in order of size from left to right and top to bottom. An interactive version of this figure may be viewed at https://genome-note-higlass.tol.sanger.ac.uk/l/?d=FAZ9iekhQ6-CeJrt7SmoIg.

The final assembly has a total length of 891.3 Mb in 136 sequence scaffolds with a scaffold N50 of 53.0 Mb ( Table 1). The snailplot in Figure 2 provides a summary of the assembly statistics, while the distribution of assembly scaffolds on GC proportion and coverage is shown in Figure 3. The cumulative assembly plot in Figure 4 shows curves for subsets of scaffolds assigned to different phyla. Most (98.32%) of the assembly sequence was assigned to 16 chromosomal-level scaffolds, representing 14 autosomes and the X and Y sex chromosomes. Chromosome-scale scaffolds confirmed by the Hi-C data are named in order of size ( Figure 5; Table 2). The following regions of this assembly are of undetermined order and orientation: Chromosome 9 region 37.4 Mbp to the end, and the whole Y chromosome. While not fully phased, the assembly deposited is of one haplotype. Contigs corresponding to the second haplotype have also been deposited. The mitochondrial genome was also assembled and can be found as a contig within the multifasta file of the genome submission.

**Table 1.** Genome data for *Lochmaea crataegi*, icLocCrat2.1.

Project accession data		
Assembly identifier	icLocCrat2.1	
Species	*Lochmaea crataegi*	
Specimen	icLocCrat2	
NCBI taxonomy ID	1143063	
BioProject	PRJEB57666	
BioSample ID	SAMEA110451579	
Isolate information	icLocCrat2, male: whole organism (DNA sequencing) icLocCrat1, whole organism (Hi-C scaffolding)	
Assembly metrics *		*Benchmark*
Consensus quality (QV)	60.4	*≥ 50*
*k*-mer completeness	100.0%	*≥ 95%*
BUSCO **	C:98.6%[S:97.2%,D:1.5%], F:0.4%,M:1.0%,n:2,124	*C ≥ 95%*
Percentage of assembly mapped to chromosomes	98.32%	*≥ 95%*
Sex chromosomes	XY	*localised homologous pairs*
Organelles	Mitochondrial genome: 18.32 kb	*complete single alleles*
Raw data accessions		
PacificBiosciences SEQUEL II	ERR10499355	
Hi-C Illumina	ERR10501013	
Genome assembly		
Assembly accession	GCA_947563755.1	
*Accession of alternate haplotype*	GCA_947563735.1	
Span (Mb)	891.3	
Number of contigs	738	
Contig N50 length (Mb)	2.8	
Number of scaffolds	136	
Scaffold N50 length (Mb)	53.0	
Longest scaffold (Mb)	139.76	

* Assembly metric benchmarks are adapted from column VGP-2020 of “Table 1: Proposed standards and metrics for defining genome assembly quality” from Rhie *et al.* (2021). ** BUSCO scores based on the endopterygota_odb10 BUSCO set using version 5.3.2. C = complete [S = single copy, D = duplicated], F = fragmented, M = missing, n = number of orthologues in comparison. A full set of BUSCO scores is available at https://blobtoolkit.genomehubs.org/view/CANOBC01/dataset/CANOBC01/busco.

**Table 2.** Chromosomal pseudomolecules in the genome assembly of Lochmaea crataegi, icLocCrat2.

INSDC accession	Chromosome	Length (Mb)	GC%
OX387423.1	1	96.32	34.0
OX387424.1	2	74.48	33.5
OX387425.1	3	54.01	33.5
OX387426.1	4	53.19	34.0
OX387427.1	5	53.02	34.0
OX387428.1	6	51.23	33.5
OX387429.1	7	47.18	34.0
OX387430.1	8	46.99	34.0
OX387431.1	9	45.26	34.5
OX387432.1	10	42.22	33.5
OX387433.1	11	41.92	34.0
OX387434.1	12	39.66	34.0
OX387435.1	13	38.78	34.0
OX387436.1	14	35.63	34.0
OX387422.1	X	139.76	34.0
OX387437.1	Y	16.76	34.0
OX387438.1	MT	0.02	22.0

The estimated Quality Value (QV) of the final assembly is 60.4 with *k*-mer completeness of 100.0%, and the assembly has a BUSCO v5.3.2 completeness of 98.6% (single = 97.2%, duplicated = 1.5%), using the endopterygota_odb10 reference set ( *n* = 2,124).

Metadata for specimens, barcode results, spectra estimates, sequencing runs, contaminants and pre-curation assembly statistics are given at https://links.tol.sanger.ac.uk/species/1143063.

## Methods

### Sample acquisition and nucleic acid extraction

A male *Lochmaea crataegi* (specimen ID Ox002129, ToLID icLocCrat2) was collected from Wytham Woods, Oxfordshire (biological vice-county Berkshire), UK (latitude 51.77, longitude –1.34) on 2022-04-28 by beating. The specimen was collected and identified by Liam Crowley (University of Oxford). The specimen used for Hi-C sequencing (specimen ID Ox001430, ToLID icLocCrat1) was collected from the same location on 2021-05-25 by beating. The specimen was collected and identified by Mark Telfer (independent researcher). Both specimens were snap-frozen on dry ice.

The workflow for high molecular weight (HMW) DNA extraction at the Wellcome Sanger Institute (WSI) includes a sequence of core procedures: sample preparation; sample homogenisation, DNA extraction, fragmentation, and clean-up. In sample preparation, the icLocCrat2 sample was weighed and dissected on dry ice ( Jay *et al.*, 2023). Tissue from the whole organism was homogenised using a PowerMasher II tissue disruptor ( Denton *et al.*, 2023a). HMW DNA was extracted using the Automated MagAttract v1 protocol ( Sheerin *et al.*, 2023). DNA was sheared into an average fragment size of 12–20 kb in a Megaruptor 3 system with speed setting 30 ( Todorovic *et al.*, 2023). Sheared DNA was purified by solid-phase reversible immobilisation ( Strickland *et al.*, 2023): in brief, the method employs a 1.8X ratio of AMPure PB beads to sample to eliminate shorter fragments and concentrate the DNA. The concentration of the sheared and purified DNA was assessed using a Nanodrop spectrophotometer and Qubit Fluorometer and Qubit dsDNA High Sensitivity Assay kit. Fragment size distribution was evaluated by running the sample on the FemtoPulse system.

Protocols developed by the WSI Tree of Life laboratory are publicly available on protocols.io ( Denton *et al.*, 2023b). 

### Sequencing

Pacific Biosciences HiFi circular consensus DNA sequencing libraries were constructed according to the manufacturers’ instructions. DNA sequencing was performed by the Scientific Operations core at the WSI on a Pacific Biosciences SEQUEL II instrument. Hi-C data were also generated from the whole organism tissue of icLocCrat1 using the Arima2 kit and sequenced on the Illumina NovaSeq 6000 instrument.

### Genome assembly, curation and evaluation

Assembly was carried out with Hifiasm ( Cheng *et al.*, 2021) and haplotypic duplication was identified and removed with purge_dups ( Guan *et al.*, 2020). The assembly was then scaffolded with Hi-C data ( Rao *et al.*, 2014) using YaHS ( Zhou *et al.*, 2023). The assembly was checked for contamination and corrected as described previously ( Howe *et al.*, 2021). Manual curation was performed using HiGlass ( Kerpedjiev *et al.*, 2018) and Pretext ( Harry, 2022). The mitochondrial genome was assembled using MitoHiFi ( Uliano-Silva *et al.*, 2023), which runs MitoFinder ( Allio *et al.*, 2020) or MITOS ( Bernt *et al.*, 2013) and uses these annotations to select the final mitochondrial contig and to ensure the general quality of the sequence.

A Hi-C map for the final assembly was produced using bwa-mem2 ( Vasimuddin *et al.*, 2019) in the Cooler file format ( Abdennur & Mirny, 2020). To assess the assembly metrics, the *k*-mer completeness and QV consensus quality values were calculated in Merqury ( Rhie *et al.*, 2020). This work was done using Nextflow ( Di Tommaso *et al.*, 2017) DSL2 pipelines “sanger-tol/readmapping” ( Surana *et al.*, 2023a) and “sanger-tol/genomenote” ( Surana *et al.*, 2023b). The genome was analysed within the BlobToolKit environment ( Challis *et al.*, 2020) and BUSCO scores ( Manni *et al.*, 2021; Simão *et al.*, 2015) were calculated.

Table 3 contains a list of relevant software tool versions and sources.

**Table 3.** Software tools: versions and sources.

Software tool	Version	Source
BlobToolKit	4.1.7	https://github.com/blobtoolkit/ blobtoolkit
BUSCO	5.3.2	https://gitlab.com/ezlab/busco
Hifiasm	0.16.1-r375	https://github.com/chhylp123/ hifiasm
HiGlass	1.11.6	https://github.com/higlass/higlass
Merqury	MerquryFK	https://github.com/thegenemyers/ MERQURY.FK
MitoHiFi	2	https://github.com/marcelauliano/ MitoHiFi
PretextView	0.2	https://github.com/wtsi-hpag/ PretextView
purge_dups	1.2.3	https://github.com/dfguan/purge_ dups
sanger-tol/ genomenote	v1.0	https://github.com/sanger-tol/ genomenote
sanger-tol/ readmapping	1.1.0	https://github.com/sanger-tol/ readmapping/tree/1.1.0
YaHS	1.1a.2	https://github.com/c-zhou/yahs

### Wellcome Sanger Institute – Legal and Governance

The materials that have contributed to this genome note have been supplied by a Darwin Tree of Life Partner. The submission of materials by a Darwin Tree of Life Partner is subject to the **‘Darwin Tree of Life Project Sampling Code of Practice’**, which can be found in full on the Darwin Tree of Life website here. By agreeing with and signing up to the Sampling Code of Practice, the Darwin Tree of Life Partner agrees they will meet the legal and ethical requirements and standards set out within this document in respect of all samples acquired for, and supplied to, the Darwin Tree of Life Project.

Further, the Wellcome Sanger Institute employs a process whereby due diligence is carried out proportionate to the nature of the materials themselves, and the circumstances under which they have been/are to be collected and provided for use. The purpose of this is to address and mitigate any potential legal and/or ethical implications of receipt and use of the materials as part of the research project, and to ensure that in doing so we align with best practice wherever possible. The overarching areas of consideration are:

- Ethical review of provenance and sourcing of the material
- Legality of collection, transfer and use (national and international)

Each transfer of samples is further undertaken according to a Research Collaboration Agreement or Material Transfer Agreement entered into by the Darwin Tree of Life Partner, Genome Research Limited (operating as the Wellcome Sanger Institute), and in some circumstances other Darwin Tree of Life collaborators.

### Data availability

European Nucleotide Archive: *Lochmaea crataegi* (hawthorn leaf beetle). Accession number PRJEB57666; https://identifiers.org/ena.embl/PRJEB57666 ( Wellcome Sanger Institute, 2022). The genome sequence is released openly for reuse. The *Lochmaea crataegi* genome sequencing initiative is part of the Darwin Tree of Life (DToL) project. All raw sequence data and the assembly have been deposited in INSDC databases. The genome will be annotated using available RNA-Seq data and presented through the Ensembl pipeline at the European Bioinformatics Institute. Raw data and assembly accession identifiers are reported in Table 1.

### Author information

Members of the University of Oxford and Wytham Woods Genome Acquisition Lab are listed here: https://doi.org/10.5281/zenodo.7125292.

Members of the Darwin Tree of Life Barcoding collective are listed here: https://doi.org/10.5281/zenodo.4893703.

Members of the Wellcome Sanger Institute Tree of Life Management, Samples and Laboratory team are listed here: https://doi.org/10.5281/zenodo.10066175.

Members of Wellcome Sanger Institute Scientific Operations: Sequencing Operations are listed here: https://doi.org/10.5281/zenodo.10043364.

Members of the Wellcome Sanger Institute Tree of Life Core Informatics team are listed here: https://doi.org/10.5281/zenodo.10066637.

Members of the Tree of Life Core Informatics collective are listed here: https://doi.org/10.5281/zenodo.5013541.

Members of the Darwin Tree of Life Consortium are listed here: https://doi.org/10.5281/zenodo.4783558.
