# Supplementary material for: The genome sequence of the hawthorn leaf beetle, Lochmaea crataegi (Forster, 1771)
Source: Wellcome Open Res. 2024 Feb 19;9:80. [Version 1] doi: 10.12688/wellcomeopenres.20911.1 (PMC11380073; doi:10.12688/wellcomeopenres.20911.1)
Supplement: Peer review history

# Reviewer report: Reviewer response for version 1

**DOI:** 10.21956/wellcomeopenres.23137.r94654

**Published:** 2024-09-06

**Authors:** Jan Veenstra

**Affiliations:** 1 University of Bordeaux, Pessac, France

Yet another high quality genome assembly from the Wellcome Sanger Institute. Having analyzed several Coleoptera genome assemblies in the past, I downloaded this one and looked for genes coding insulin and IGF related ligands and their putative receptors. As I expected there was no problem identifying these genes and predicting their coding exons. Thus confirming, for admittedly a relatively small number of genes, the supreme quality of this gene assembly.

Are sufficient details of methods and materials provided to allow replication by others?

Yes

Is the rationale for creating the dataset(s) clearly described?

Yes

Are the datasets clearly presented in a useable and accessible format?

Yes

Are the protocols appropriate and is the work technically sound?

Yes

Reviewer Expertise:

Insect neuropeptides

I confirm that I have read this submission and believe that I have an appropriate level of expertise to confirm that it is of an acceptable scientific standard.

# Reviewer report: Reviewer response for version 1

**DOI:** 10.21956/wellcomeopenres.23137.r87812

**Published:** 2024-07-22

**Authors:** Manee M Manee

**Affiliations:** 1 King Abdulaziz City for Science and Technology, Riyadh, Saudi Arabia

This study presents a comprehensive genome assembly for the hawthorn leaf beetle. The assembly quality metrics are robust, with an estimated Quality Value (QV) of 60.4, k-mer completeness of 100.0%, and a BUSCO completeness of 98.6%. The study employs a rigorous methodology, using advanced sequencing and assembly techniques. Detailed descriptions of sample collection, DNA extraction, sequencing, and assembly provide transparency and reproducibility. I am happy with the current form of this study.

Are sufficient details of methods and materials provided to allow replication by others?

Yes

Is the rationale for creating the dataset(s) clearly described?

Yes

Are the datasets clearly presented in a useable and accessible format?

Yes

Are the protocols appropriate and is the work technically sound?

Yes

Reviewer Expertise:

bioinformatics, genomics and evolutionary biology

I confirm that I have read this submission and believe that I have an appropriate level of expertise to confirm that it is of an acceptable scientific standard.
